# Prevalence and Correlates of Alcohol Consumption among Hill-Tribe Adolescents below the Legal Drinking Age—A Community-based Cross-Sectional Study in Northern Thailand

**DOI:** 10.3390/ijerph17218266

**Published:** 2020-11-09

**Authors:** Narongsak Noosorn, Civilaiz Wanaratwichit, Shamsudeen YAU, Nuansiri Kedsai

**Affiliations:** Department of Community Health, Faculty of Public Health, Naresuan University, Phitsanulok 65000, Thailand; civilaiz_6@hotmail.com (C.W.); sybirninkudu2013@gmail.com (S.Y.); drsane2513@gmail.com (N.K.)

**Keywords:** alcohol, prevalence, correlates, adolescent, hill tribe, northern Thailand

## Abstract

Alcohol drinking has been prevalent among various hill tribes of northern Thailand due to their distinctively different lifestyles, traditions, cultures and beliefs than the general Thai population; the majority of these traditions involve annual rounds of customary rituals that make alcohol abundantly accessible to all age groups. To study the prevalence and predictors of alcohol use, a community-based analytical cross-sectional study was conducted between October 2017–September 2018 among 480 sampled adolescents aged 12 through 18 years drawn by stratified random sampling. A proprietary questionnaire developed by the researchers was used to collect the data which were analyzed using binary logistic regression. The prevalence of alcohol use in the previous 30 days was 46.7%. Drinking predictors were: having at least a drinking parent, drinking peers, ever been sent to buy alcohol, smoking, cordial relationship with peers, gambling, family violence experience, stress and leisure time company. Therefore, our results suggest that prevention interventions should be designed with a focus on discouraging parents from sending children to buy alcohol and drinking or smoking in their presence; to reduce both social and commercial access to alcohol, age limits should be imposed on alcohol intake at all ceremonious events, while strictly reinforcing the law that prohibits selling alcohol to minors.

## 1. Introduction

Alcohol consumption causes significant health and economic consequences which could be chronic and/or acute, resulting from mortality, morbidity and disability. Over the decades, alcohol-related research has continued to take interest in the variability of the impact of the overall consumption and patterns. Alcohol consumption among both adult and adolescent populations in Thailand has been both a public health and social problem in the country for decades. Although Thailand has robust alcohol control policies, which quite adequately incorporate all relevant regulatory sectors to explicitly address alcohol-related problems, effective implementation of these policies, which requires smooth interactions between those regulatory sectors, has been a major setback to controlling alcohol access and availability [1]. One of these policies, the Thai Alcoholic Beverage Control Act (2008), has univocally and categorically refuted selling alcohol to persons younger than twenty years [2]. Yet, the prevalence of alcohol consumption continues to rise, and it varies from one subset of the population to another. 

A 58% prevalence, for instance, was reported among university students [3], 22% among school-attending adolescents [4] who are below the legal drinking age, and 5.6% in pregnant women [5]. The overall national prevalence among Thais’ 15 years and older had increased from 28.6% in 2007 [6] to between 32–36% in the currently elapsing decade [7]. This translates into 8.3 L of pure alcohol per capita consumption (APC), which represents the highest APC in the entire south-east Asia region [8]. Until recently, these figures had been on a consistent rise, with periodic small fluctuations, decade after decade. The latest projection suggests that by the year 2025, the APC will rise even higher [8]. Not surprisingly, the prevalence of alcohol-related disorders in Thailand doubled the average in the south-east Asia region as reported in 2010 [8,9]. In addition, both mild and severe intentional and unintentional injuries, risky sexual behaviors, tobacco use, prescription and illicit drugs use and misuse among the general Thai youths have been connected to alcohol use [10,11]. Furthermore, higher BMI in university students [3], road traffic accidents [12], non-communicable diseases [13] and other health-risk behaviors [14] have been associated with alcohol. Additionally, tendencies for juvenile delinquency are also associated with alcohol as 40% of crimes perpetrated by Thai youths involves alcohol, reportedly [8]. Factors associated with adolescent alcohol consumption include engaging in violent activities, relationship violence, contemplating suicide, being sexually active and having ever impregnated someone [10]. 

Northern Thailand region has been characterized by the highest alcohol consumption rates. A study of the Provincial Alcohol Index (PAI) showed that northern provinces have the highest PAI score as opposed to other regions [15]. Nearly a quarter of Thailand’s 76 provinces are located in the northern region. In most of these provinces, especially those in the lower north, significant areas have a characteristically mountainous landscape, which are homes to various tribal populations. Hmong, Lahu, Lishu, Yao, etc. are a few examples of the tribes living in these highland areas. These hill tribes have distinctively different lifestyles, traditions, cultures and beliefs than the general Thai population [16]. The majority of these traditions involve annual rounds of customary rituals that make alcohol abundantly accessible to all age groups. For instance, alcohol is traditionally served to everyone during the Hmong’s New Year celebration, which is usually filled with cheerfulness and long-preserved rituals where descendants show their gratitude towards their ancestors. Other traditions at which alcohol is served are wedding ceremonies and rice harvest celebrations.

Not surprisingly, the prevalence of alcohol use among youths (15–24 years) of these tribes was 45%, with factors such as being male gender, Buddhist, employment and unemployment status, having a father as a wage earner, parents in an agricultural occupation, having an alcohol-using father and having six or more friends who use alcohol [11,17,18]. Given this high prevalence of alcohol consumption among the hill tribes, it is imperative to study and understand the dynamics of consumption among their adolescent population below the legal drinking age. Therefore, the primary objective of this study was to examine the prevalence and predicting factors of alcohol use among adolescents of highland tribes. This could inform the designing of future prevention interventions and improving the existing ones.

### Definitions of Some Variables

The conceptual definitions of some of the sociocultural, socio-demographic and other variables are given below.

Sociocultural variables: These variables captured participants’ experiences of domestic violence, night outing, pornography, online gaming, living alone or with a friend, sexual practice, gambling and drinking in traditional events.

Knowledge variable: This variable measured participants’ knowledge of alcohol-related consequences such as road traffic accident, physical and mental health problems, possible drug use and criminal activities and alcohol laws for minors in Thailand.

Attitude: Measured participants’ attitude towards alcohol drinking

Stress: This variable measured participants’ stress perceptions based on the extent to which situations in their lives are appraised as stressful. The adopted items were designed to predict how unpredictable, uncontrollable and overloaded respondents find their lives.

## 2. Materials and Methods 

### 2.1. Study Design, Settings and Participants

This was an analytical cross-sectional study conducted among hill-tribe adolescents in the northern region of Thailand. To ensure full representation of both in-school and out-of-school adolescents, participants from Hmong, Lahu, Lishu and Yao tribes aged 12 through 18 years, registered or unregistered in the education registry, were recruited to participate in the study between October 2017–September 2018. They comprised both genders residing in 37 village communities along the borders of three lower northern provinces—Petchabun, Tak and Phitsanulok—where the population of hill tribes is dense. Using the formula of Cochran [19] as shown below, a sample size of 384 was determined. To account for any cases of non-response, incompleteness of data, parental objection for their children’s participation in the study or any other unforeseen reasons that could potentially shorten the sample size, 25% of the sample was added bringing the total to 480.

n0 = Zα22p(1−p)d2

*n*_0_ = the desired sample size from a large population size.
*z*_α/2_ = two-tailed Z-score confidence level (1.96).
*p* = Population proportion (0.5).
*d* = desired precision level (0.05)

$$n_{0\;}=\;\frac{{\left(1.96\right)}^{2}\;\times 0.5\times \left(1-0.5\right)}{{\left(0.05\right)}^{2}}=384.16$$

$$\frac{25}{100}\;\times 384=96$$

384+96=480

The sample selection was carried out using stratified random sampling. First, the region’s seventeen (17) provinces were stratified into three subgroups based on their population densities—high density, medium density and low density. From each stratum, a province was randomly selected. From each selected province, a sample was randomly drawn proportionate to the population size. This was to ensure that the sample drawn was adequately representative and accurately generalizable.

### 2.2. Research Tool

A well-developed questionnaire (Supplementary Materials) containing both open-ended and close-ended questions was used as the research tool. It comprised six distinct sections, namely: (1) socio-demographics, (2) sociocultural, (3) alcohol use and related factors, (4) knowledge of consequences of alcohol, (5) attitude towards alcohol drinking and (6) life stress. Except for the stress items that were adopted from the Perceived Stress Scale [20], all other items in the questionnaire were informed based on an extensive review of previous research and it was validated for internal consistency by experts. The reliability test was carried out on 40 samples who were of comparable characteristics with the study target population, and the test produced a Cronbach’s alpha of 0.89, 0.86 and 0.84 for knowledge, attitude and stress.

Attitude and stress were measured on a 5-point Likert scale. Attitude scale ranged from ‘strongly agree’ (score = 5) to ‘strongly disagree’ (score = 1) while stress scale ranged from ‘never’ (score = 0 for negative questions or 4 for positive questions) to ‘often’ (score = 4 for negative questions or 0 for positive questions). Knowledge was measured on a 3-point scale (3; agree–1; disagree). There were ten questions each for attitude, knowledge and stress. Therefore, the possible range of scores was 10–50 points for attitude, 10–30 points for knowledge and 0–40 for stress. Each participants’ score was then categorized into three levels—low, moderate and high—using the Best’s criteria for attitude and stress and Bloom’s criteria for knowledge.

In the exploratory factor analysis (EFA), the principal component was performed on the 10 items of attitude toward drinking. The factors were subjected to orthogonal (varimax) rotation to maximize the dispersion of the loadings within factors so that loading a smaller number of variables highly onto each factor results in more interpretable clusters of factors. Factor analysis and scree test showed that a five-factor was most appropriate. The five factors accounted for 81.11% of the variance in scores. Factor 1 accounted for 20.06% of the variance, factor II accounted for 19.64%, factor III for 18.69%, factor IV for 12.18% and factor V accounted for 10.53% of the variance.

### 2.3. Data Collection

The data collection process was carried out in the hill tribe communities situated at the borders of three selected provinces. The questionnaire was self-administered to the participants who were allowed to respond at their own pace. The respondents were allowed enough time to respond to the questions to minimize recall bias while recollecting past events. The process was facilitated by three research assistants with years of experience in field surveys. In total, 480 questionnaires were retrieved fully filled making the participatory rate 100%.

### 2.4. Data Analysis

The data were analyzed using a statistical software program (SPSS^®^, version 22.0, New York, NY, USA). Categorical variables were presented as frequencies and percentages using descriptive statistics. A univariate analysis was used to examine the unadjusted association between alcohol drinking and sociodemographic, sociocultural, knowledge, attitude and stress variables; two-tailed Pearson chi-squared tests were utilized to determine the statistical significance. As a measure of the magnitude of the association between the independent variables (sociodemographic, sociocultural, knowledge, attitude and stress) and our dependent variable of interest (alcohol drinking), odds ratios (ORs) and 95% confidence intervals (95% CIs) were produced using binary logistic regression (BLR). Essentially, only variables associated with alcohol drinking at a 25% or lower statistical significance in the unadjusted univariate analysis were qualified and included in the adjusted multivariate analysis. All analyses, except otherwise indicated, were performed at a 5% level of statistical significance with a 95% level of confidence. 

### 2.5. Ethical Certification

The research was conducted in full accordance with the Declaration of Helsinki and was approved by the Institutional Review Board (IRB) of Naresuan University, Thailand. The project’s certificate of approval (CoA) number and the IRB number are 115/2018 and 1141/60, respectively. 

## 3. Results

As described in Table 1, nearly 69% of the participants were between 12–15 years and 31% between the ages of 16–18 (overall mean age; 13.96 ± 1.08). Over two-thirds were males, the majority (67.5%) of whom were in secondary/high schools and nearly half (47%) of them performed poorly in school (CGPA < 2.00). The Hmong, Lahu, Lishu and Yao tribes constituted, respectively, 28.3%, 25.6%, 24.0% and 22.1% of the participants. The vast majority of their parents (88%) were still married and more than half (57%) were low-income earners. Nearly three-quarters (73%) of the participants frequented temples for religious worships and events. While a large proportion (78%) of the participants had a medium knowledge of the negative consequences of alcohol consumption, the overwhelming majority (83%) had a liberal attitude towards drinking alcohol. 

The prevalence of alcohol consumption 30 days preceding the survey, as presented in Table 2, was 46.7%. As clearly indicated in Table 3, the overwhelming number of them drank at one type of annual or ceremonious event or another. Of those, 51% drank at their friends’ houses, nearly 46% in their homes and 2% in students’ dormitory (Table 4). While 95% of the participants had been sent to buy alcohol at least once in their lifetime, more than three-quarters (77%) had at least a parent who drinks alcohol at home (Table 2). The vast majority (90%) spent their leisure time in the company of their friends, of whom 72% were drinkers. Most of the participants (88%) were either living alone or with friends whom they maintained a cordial relationship with the overwhelming majority (77%). Nearly 12% and 26% of them engaged in gambling and violence-related activities, respectively. The stress level was medium for most of the participants (96.5%). 

Table 5 depicts the univariate relationship between alcohol drinking and other variables. It can be seen that among the several variables associated with alcohol drinking, gender, drinking parents, drinking peers, smoking, gambling and family violence were strongly associated with drinking alcohol (*p*-value < 0.001). Other associated variables were academic performance, leisure time company, relationship with peers and ever sent to buy alcohol (*p*-value < 0.05).

### Predictors of Alcohol Consumption

The multivariate analysis showed that alcohol drinking among adolescents was multifactorial. It can be seen in Table 6 that adolescents whose parents drink alcohol at home were two and half times more likely to drink alcohol than those whose parents did not drink (OR = 2.51 (1.22–5.19), *p*-value = 0.013). This implies that having parents who drink alcohol at home is strongly associated with the risk of alcohol drinking. Additionally, adolescents whose peers are reported to drink were at a nearly 14-fold risk of alcohol consumption than those whose peers were non-drinkers (OR = 13.63 (5.68–32.69), *p*-value = <0.001). Equally, the risk increases by approximately seven times among adolescents who had ever been sent to buy alcohol than their counterparts who had not (OR = 6.55 (1.07–40.03), *p*-value = 0.042). Similarly, smoking was associated with alcohol intake as smokers were more than three times as likely to consume alcohol as non-smokers (OR = 3.38 (1.53–7.47), *p*-value = 0.003). Furthermore, adolescents who maintained a cordial relationship with their peers were at increased risk of alcohol consumption (OR = 8.47 (3.62–19.79), *p*-value = 0.001). This means that adolescents who were more friendly with their peers were more than eight times as likely to drink alcohol as those in a non-cordial relationship. Further, gambling adolescents were at four and a half times the risk of alcohol consumption than those who do not gamble (OR = 4.46 (1.16–12.44), *p*-value = 0.004). In addition, those who reported having experienced family violence were nearly three times more likely to drink alcohol compared to those who did not (OR = 2.69 (1.42–5.10), *p*-value = 0.002). The stress level was also a predicting factor for alcohol consumption. It was found that adolescents at a high level of stress were more than twice as likely to drink alcohol than those with a moderate stress level (OR = 2.53 (1.48–4.34), *p*-value = 0.001). Lastly, adolescents who spent their leisure time in the company of their friends were nearly eighty percent less likely to drink than those in the company of their parents (OR = 0.19 (0.08–0.50), *p*-value = 0.001). 

## 4. Discussion

The population studied was among the age group whom the Thai alcohol law has unambiguously forbidden from both drinking and purchasing all kinds of alcohol. However, the uncharacteristic cultural and traditional settings of the hill tribes have made alcohol access to these youths so easy. With a disturbing prevalence of 46.7%, it is arguable to imply that the adolescents of Hmong, Lahu, Lishu and Yao hill tribes are vulnerable to dangerous consumption of alcohol. This is more than twice the national average prevalence (22.2%) in general Thai youths of the same age group [4]. This finding agrees with a recent study among youths of Akha and Lahu tribes 15–24 years, which revealed a 45% prevalence of alcohol use. Contrastingly, unlike the present study that suggested females were a greater proportion of drinkers, the study also reported a higher proportion of alcohol users among males [18]. This difference could be partly explained by the cultural differences between the tribes that participated in both studies, and largely by the possibility that our study might have overly sampled female drinkers. Although the participants of both studies, who were from the six dominant tribal groups in the northern region (Akha, Hmong, Lahu, Lishu, Yao and Karen), were originally from southern China. Years after migration, some of them still held onto their ancestral distinct lifestyles of social interaction more tightly than others given rise to variants of cultures.

Furthermore, the study found that adolescents whose one or both parents drink alcohol were more likely to use alcohol. This coincides with well documented evidence suggesting that children born to alcoholic parents are at increased risk of alcohol use and addiction [21,22,23,24], and it has also been reported among other hill tribes [18] and female students [17]. This may not be unconnected to the fact that children see their parents as role models and, therefore, imitate what the parents do. Consequently, as they grow into adolescence, they tend to put whatever they had learned into practice with little or no regards for its consequences. In addition, group drinking is considered an important lifestyle of the Hmong tribes in Khao Koh community. Members of this community believe that group drinking within a family brings success, strengthens family ties, relieves fatigue and tiredness, boosts appetite and energy to work. This has made drinking customary to this community and offering alcohol to individuals, regardless of their age, is considered a symbol of kindness. This communal practice particularly has become a distinguishable social value that children and adolescents have become accustomed to seeing adults regularly drink alcohol. This could downplay their perception of alcohol drinking; they may perceive nothing is wrong with it as it represents the picture they generally see in the community. Consequently, drinking has become an indistinguishable lifestyle of the Hmong tribe. 

Not surprisingly, keeping peers who were alcohol users was highly significantly associated with increased risk of alcohol use. Coincidently, a recent study among older Akha and Lahu youths suggested that nearly half of the participants started drinking due to peer group persuasion [18]. Furthermore, similar findings in Thai vocational school students [25], high school female students [17] and US adolescents and youths reported the same association [26]. Arguably, the extent and dimensions to which peer pressure exerts influence on alcohol drinking among school adolescents have been thoroughly discussed [27]. However, this might be attributed to a misperception of higher drinking by peers, which has been shown to drive higher consumption by school-age adolescents [28]. Worthy of note is that the hill tribe communities are usually remote and small making interactions between adolescents relatively easy, which allows peers bonding to one another and thereby learning and unlearning one another’s behaviors. This explains why adolescents who maintain a cordial friendship with their peers were more likely to drink than those in a non-cordial relationship. Moreover, the majority of the parents travel a long distance to work in farmlands and, sometimes, stay there for days. Until the parents return, the kids, who are usually many as the hill tribe people are known for their high parity, are left alone to take care of each other on their own. This could lead to poor parental connectedness and guidance, which has been reported to increase the risk of alcohol use among general Thai adolescents [4,29].

Against the provision of the law, as enshrined in the Thai Alcoholic Beverage Control Act [2], which prohibits selling alcohol to minors, Thai parents still send their underage children to buy alcohol from the local grocery stores. An overwhelming proportion of the participants acknowledged that they had at least once been sent to buy alcohol. Worryingly, this significantly increases the adolescents’ risk of alcohol use by many folds. Evidence has shown that Thai vocational school students who could purchase alcohol from alcohol outlets were more likely to drink alcohol or binge drink in the previous 30 days [25]. Not only the parents, but many alcohol retailers also defy the law and sell alcoholic products to adolescents. A study in Chiang Mai adolescents reported that it is easy to purchase alcohol from retailers in the neighborhood who are keen to maximize profit in their business [30]. This social availability of alcohol, which has continued to give adolescents easy access to the product, has greatly contributed to consumption at school age, including engaging in binge drinking [25]. 

Expectedly, we found that adolescent smokers were more likely than non-smokers to consume alcohol. Coincidently, a documented body of evidence among Benin [31] and Thai adolescents [4,29] reported that, for both genders, adolescent tobacco use is a strong predictor of subsequent alcohol use. Another separate study of freshmen students reported that current smokers were more likely to frequently drink, to engage in binge drinking and to present multiple physiological repercussions [32]. This is unsurprising as mounting evidence of co-occurrence of adolescent health risk behaviors such as smoking and alcohol; family violence and alcohol, smoking and sexual risk behavior and alcohol and drug use have been well documented [33,34,35]. Similar to alcohol, tobacco use among the hill tribe people is yet another common practice of important public health concern. Therefore, in addition to alcohol, adolescents could have learned tobacco use from their parents/guardians as they gather to drink in groups. Further, for what seemed to be a purpose of convenience, hill tribe still send their children to buy cigarettes from the local stores, which exposes them to the tobacco products. Similarly, the risk of drinking was significantly higher among adolescents who reported experience of family violence than those who did not. This corroborates with the findings among Brazilian adolescents in whom family violence was reported to influence adoption and co-occurrence of risk behavior such as alcohol consumption [35]. Consequently, alcohol consumption has been implicated in increasing adolescent violence and crimes [15]. According to the WHO, 4 in 10 adolescent crimes in Thailand involve alcohol [8] but are surprisingly more pronounced among girls than boys [36].

Susceptibility to alcohol appeared to be higher among gambling adolescents than their non-gambling counterparts; the risk was nearly four and a half times. This was consistent with several studies done over the decades which have demonstrably elaborated the association between adolescent alcohol consumption and gambling among populations of different races, ages and educational attainments [37,38,39,40]. For instance, having gambling families or peers [39], type of gambling mates [40], gambling frequency [38] and adolescent delinquency [37] have all been associated with alcohol use. Given that gambling is usually associated with anxiety which could expectedly cause serious distress, it is not surprising that the gambling adolescents in the present study showed increased risks of alcohol consumption. Understandably, it may be safe to assume that they resort to taking alcohol as a coping strategy to relieve themselves of the anxiety and the losses that come with the gambling.

The study also discovered that high stressed adolescents were more likely to drink than those whose stress level was moderate. Concordant to this finding was a study among Hispanic adolescents which suggested that higher stress scores were noted among youths who admitted alcohol use in the preceding 30 days [41]. A similar trend was observed among Grade 12 students, but the severity of the effect of stress on alcohol intake appeared to be buffered by positive peer influence [42]. Given that the overwhelming majority of the hill tribes are farmers whose children usually assist them with work in the farmlands, it is understandable to have high-stress levels among the adolescents, which makes stress-induced alcohol intake highly possible. Moreover, it has been a common practice to gather around the house and drink in the evening as a way of relieving work stress and tiredness. Fundamentally, stress induces alcohol intake, but the understanding of how this pathway works is limited to only a few biomarkers and biological processes [43], which calls for more research for further understanding of it. Lastly but unexpectedly, the study found that the type of company the adolescents spend their leisure time with is a significant predictor of alcohol consumption. Despite admitting that more than two-thirds of their peers used alcohol, surprisingly, spending leisure time with peers rather than parents appeared to significantly reduce the risk of alcohol consumption. Although having drinking peers has been repeatedly associated with increased risk of drinking [17,18,25,26], positive peer influence reduces the risk [42]. Therefore, it may imply that our participants’ peers had a positive attitude towards drinking which may discourage alcohol use. 

## 5. Conclusions

Nearly 5 in 10 hill-tribe adolescents have used alcohol; more than 9 in 10 had been sent to buy alcohol at least once in their lifetime; almost 8 in 10 had at least a drinking parent; 7 in 10 reported having a drinking peer. Therefore, prevention interventions should be designed with a focus on discouraging parents from sending children to buy alcohol and drinking or smoking in their presence. To reduce both social and commercial access to alcohol, age limits should be imposed on alcohol intake at all ceremonious events, while strictly reinforcing, on alcohol retailers, the law that prohibits selling alcohol to minors. 

In conclusion, the data in this study showed that all the female participants reported drinking alcohol in the previous 30 days. Thus, we could not separately examine the potential gender difference regarding the predictors of alcohol consumption. Therefore, it is strongly recommended that future research should explore gender influence on alcohol drinking behaviors among this cohort. 

## Figures and Tables

**Table 1:** General characteristics.

Variables	*n* (%)		
	Males (*n* = 315)	Females (*n* = 165)	Total (*n* = 480)
Age group (years)			
12–15	220 (69.8)	110 (66.7)	330 (68.8)
16–18	95 (30.2)	55 (33.3)	150 (31.2)
Gender			
Male	-	-	315 (65.6)
Female	-	-	165 (34.4)
Tribe			
Hmong	90 (28.6)	46 (27.9)	136 (28.3)
Lahu	87 (27.6)	36 (21.8)	123 (25.6)
Lishu	65 (20.6)	50 (30.3)	115 (24.0)
Yao	73 (23.2)	33 (20.0)	106 (22.1)
Education level			
Primary school or lower	70 (22.2)	53 (32.2)	126 (25.6)
Secondary/high school	220 (69.8)	106 (63.0)	324 (67.5)
Vocational school	25 (7.0)	8 (4.8)	33 (6.9)
Parental marital status			
Single/widowed/separated	38 (12.1)	19 (11.5)	57 (11.9)
Married	277 (87.9)	146 (88.5)	423 (88.1)
Academic Performance (CGPA)			
Poor (<2.00)	136 (43.2)	89 (53.9)	225 (46.9)
Good (≥2.00)	179 (56.8)	76 (46.1)	255 (53.1)
Parental income (Baht)			
Low (Below 9000)	140 (44.4)	135 (81.8)	275 (57.3)
Medium (9001–30000)	175 (55.6)	30 (18.2)	205 (42.7)
Going to temple			
Always	227 (72.1)	124 (75.2)	351 (73.1)
Rarely	88 (27.9)	41 (24.8)	129 (26.9)
Exercise			
Always	60 (19.0)	39 (23.6)	99 (20.6)
Rarely	255 (81.0)	126 (76.4)	381 (79.4)
Knowledge			
Low	55 (17.5)	41 (24.8)	96 (20.0)
Medium	253 (80.3)	123 (74.5)	376 (78.3)
High	7 (2.2)	1 (0.6)	8 (1.7)
Attitude			
Medium	51 (16.2)	30 (18.2)	81 (16.9)
High	264 (83.8)	135 (81.8)	399 (83.1)

**Table 2:** Prevalence of alcohol consumption and other determining factors (*n* = 480).

Variables	*n* (%)		
	Males (*n* = 315)	Females (*n* = 165)	Total (*n* = 480)
Drinking status			
Yes	59 (18.7)	165 (100)	224 (46.7)
No	256 (81.3)	-	256 (53.3)
Leisure time company			
Parents	31 (9.8)	16 (9.7)	47 (9.8)
Friends	284 (90.2)	149 (90.3)	433 (90.2)
One or both parents drink alcohol at home			
Yes	231 (73.3)	140 (84.8)	371 (77.3)
No	84 (26.7)	25 (15.2)	109 (22.7)
Sent to buy alcohol			
Yes	295 (93.7)	160 (97.0)	455 (94.8)
No	20 (6.3)	5 (3.0)	25 (5.2)
My peers drink alcohol			
Yes	204 (69.8)	139 (84.2)	343 (71.5)
No	111 (35.2)	26 (15.8)	137 (28.5)
Smoking Status			
Yes	42 (13.3)	-	42 (8.8)
No	273 (86.7)	160 (100.0)	438 (91.2)
Living with			
Family (mum/dad/relatives)	32 (10.2)	27 (16.4)	59 (12.3)
Alone/friend	283 (89.8)	138 (38.6)	421 (87.7)
Relationship with friends			
Cordial	59 (18.7)	52 (31.5)	111 (23.1)
Non-cordial	256 (81.3)	113 (68.5)	369 (76.9)
Gambling Status			
Yes	10 (3.2)	47 (28.5)	57 (11.9)
No	305 (96.8)	118 (71.5)	423 (88.1)
Family violence			
Yes	57 (18.1)	67 (40.6)	124 (25.8)
No	258 (81.9)	98 (59.4)	356 (74.2)
Stress			
Medium	302 (95.9)	161 (97.6)	463 (96.5)
High	13 (4.1)	4 (2.4)	17 (3.5)

**Table 3:** Proportions of adolescents who drink at ceremonious cultural events (*n* = 224).

Drinking Relevant to Culture	Yes (%)	No (%)
1. Ever drank at a traditional event?	224 (100.0)	0 (0.0)
2. Ever served alcohol at a traditional event?	210 (93.7)	14 (6.3)
3. Ever drank during a new year ceremony?	224 (100.0)	0 (0.0)
4. Ever drank at a wedding ceremony event?	224 (100.0)	0 (0.0)
5. Ever drank at an annual harvest festival?	199 (88.8)	25 (11.2)
6. Ever drank at a religious event?	200 (89.3)	24 (10.7)
7. Are there alcohol shop near your house?	177 (79.0)	47 (21.0)
8. Are there alcohol shop near your school?	187 (83.5)	37 (16.5)

**Table 4:** Participants’ drinking venues (*n* = 224).

Venue	*n* (%)
At home	104 (46.4)
Friend’s house	115 (51.3)
Student’s dormitory	5 (2.2)

**Table 5:** Univariate relationship between alcohol drinking and other variables (*n* = 224).

Variables	n (%)	X ^2^	df	γ	*p*-Value
Gender					
Male	59 (18.7)	287.347	1	−0.774	<0.001 *
Female	165 (100)				
Age group (years)					
12–15	161 (48.8)	1.909	1	−0.063	0.167
16–18	63 (42.0)				
Education level					
Primary school or lower	66 (53.7)	3.594	2	−0.087	0.166
Secondary/high school	145 (44.8)				
Vocational school	13 (39.4)				
Parental marital status					
Single/widowed/separated	27 (47.4)	0.013	1	−0.005	0.910
Married	197 (46.6)				
Academic Performance (CGPA)					
Poor (<2.00)	119 (52.9)	6.588	1	−0.117	0.010 *
Good (≥2.00)	105 (41.2)				
Leisure time company					
Parents	33 (14.7)	11.606	1	−0.155	0.001 *
Friends	191 (853)				
One or both parents drink alcohol at home					
Yes	192 (51.8)	16.976	1	0.188	<0.001 **
No	32 (29.4)				
Living with					
Family (mum/dad/relatives)	31 (52.5)	0.933	1	−0.044	0.234
Alone/friend	193 (45.8)				
My peers drink alcohol					
Yes	189 (55.1)	34.357	1	0.268	<0.001 **
No	35 (25.5)				
Relationship with friends					
Cordial	61 (55.0)	3.985	1	0.091	0.046 *
Non-cordial	163 (44.2)				
Smoking Status					
Yes	32 (76.2)	16.120	1	0.183	<0.001 *
No	192 (43.8)				
Sent to buy alcohol					
Yes	219 (48.1)	7.535	1	0.125	0.006 *
No	5 (20.0)				
Gambling Status					
Yes	47 (82.5)	33.287	1	0.263	<0.001 **
No	177 (41.8)				
Family violence					
Yes	81 (65.3)	23.380	1	0.221	<0.001 **
No	143 (40.2)				
Going to temple					
Always	161 (45.9)	0.334	1	−0.026	0.563
Rarely	63 (48.8)				
Exercise					
Always	53 (53.5)	2.364	1	0.070	0.124
Rarely	171 (44.9)				
Parental income (Baht)					
Low (Below 9000)	135 (49.1)	1.520	1	−0.056	0.218
Medium (9001–30000)	89 (43.4)				
Attitude					
Medium	41 (50.6)	0.611	1	−0.036	0.434
High	183 (45.9)				
Stress					
Moderate	219 (47.3)	2.108	1	−0.066	0.147
High	5 (29.4)				

* *p* < 0.05; ** *p* < 0.001

**Table 6:** Multivariate analysis of factors associated with alcohol drinking.

Variables	OR (95% C.I.)	*p*-Value
One or both parents drink alcohol at home		
No	-	-
Yes	2.51 (1.22–5.19)	0.013 *
My peers drink alcohol		
No	-	-
Yes	13.63 (5.68–32.69)	<0.001 **
Relationship with friends		
Non-cordial	-	-
Cordial	8.47 (3.62–19.79)	<0.001 **
Do you smoke?		
No	-	-
Yes	3.38 (1.53–7.47)	<0.003 *
Sent to buy alcohol		
No	-	-
Yes	6.55 (1.07–40.03)	0.042 *
Gambling Status?		
No	-	-
Yes	4.46 (1.60–12.44)	0.004 *
Family violence		
No	-	-
Yes	2.69 (1.42–5.10)	0.002 *
Stress		
Moderate	-	-
High	2.53 (1.48–4.34)	0.001 *
Leisure time company		
Parents	-	-
Friends	0.19 (0.08–0.50)	0.001 *

* *p* < 0.05; ** *p* < 0.001

## Supplementary Materials

The following are available online at https://www.mdpi.com/1660-4601/17/21/8266/s1, The research questionnaire.
