# Supplementary material for: Prevalence and Correlates of Alcohol Consumption among Hill-Tribe Adolescents below the Legal Drinking Age—A Community-based Cross-Sectional Study in Northern Thailand
Source: Int J Environ Res Public Health. 2020 Nov 9;17(21):8266. doi: 10.3390/ijerph17218266 (PMC7664938; doi:10.3390/ijerph17218266)
Supplement: Supplementary file 1 [file ijerph-17-08266-s001.pdf]

Questionnaire

Part 1 Socio-demographics

1. Address

   District...

2. Are you a student? ☐ 1. yes ☐ 2. No

3. What is your tribe group?

   ☐ 1. Hmong ☐ 2. Lahu

   ☐3. Lishu ☐ 4. Yao

4. Sex ☐ 1. Male ☐ 2. Female

5. Age……………. (in years)

6. Parental marital status

   ☐ 1. Single/widowed/separated ☐ 2. Married

   ☐ 3 Other…………………………………

7. Education level

   ☐ 1.Uneducated ☐ 2. Primary school

   ☐ 3.Secondary school ☐ 4. High school

   ☐ 5.Vocational school ☐ 6. University

8. What is your academic performance (CGPA)

   ☐ Poor (< 2.00) ☐Good (≥ 2.00)

9. Father’s occupation

□1. Farmer □2. An employee □3. Business

□4. Unemployed

□5. Company □6. Government officer □7. Other (identify)........................

10. Mother's occupation

□1. Farmer □2. An employee □3. Business

□4. Unemployed

□5. Company □6. Government officer □7. Other (identify)......................

11. What is your parental income (Baht)?

‹ 1. < 9000 ‹ 2. 9001-30000 ‹ 3. > 30000

12. How frequent do you go to the temple?

□ Always □ Rarely

13. How often do you exercise?

□ Always □ Rarely

14. What's your daily expenses (Baht)

☐ < 10 ☐ 11 – 40 ☐ 41 – 70

☐71 – 100 ☐100 – 130 □ 7 . Other (identify)...................

15. What is the size of your family?

☐ 2 – 3 people ☐ 4 – 6 people

☐ 7 – 9 people ☐ Other (identify)....................

16. Who do you stay/live with?

☐ Father and Mother ☐ Mother or Father

☐ A friend

☐ A relative ☐ Other

(identify)..

17. Residence

☐ Own house ☐ Relatives' house ☐ A dormitory

☐ A friend's house ☐ Other

(identify)................................

18. What do you do with your free time?

☐ Reading ☐ Play game online ☐ Access internet

☐ Watch TV ☐ Exercise ☐ Shopping

☐ Other (identify)..............

19. Who do you usually spend your free time with?

☐ Parents ☐ Friends

20. Do both or one of your parents drink alcohol at home?

☐ Yes ☐ No

21. Do your friends drink alcohol?

☐ Yes ☐ No

22. What is the nature of your relationship with your friends?

☐Friendly ☐Non-friendly

Part 2 Socio-cultural

Choose the option that best describes your experience of the following events

1. Violence in the family ☐ 1.Regulary ☐2.Sometimes☐ 3.Never
2. Ill treatment from a family member ☐ 1.Regulary ☐2.Sometimes ☐ 3.Never
3. Do you go to nightlife venues ☐ 1.Regulary ☐2.Sometimes ☐ 3.Never
4. Do you watch pornography ☐ 1.Regulary ☐2.Sometimes ☐ 3.Never 5. Do you play online games ☐ 1.Regulary ☐2.Sometimes ☐ 3.Never
6. Have you ever had sex? ☐ 1.Regulary ☐2.Sometime ☐ 3.Never
7. Play football betting ☐ 1.Regulary ☐2.Sometime ☐ 3.Never

Part 3 Alcohol use

a) Drinking relevant to culture

1. Have you ever taken alcohol at a traditional event?

☐1.Yes ☐ 2. No

2. Have you ever served alcohol at a traditional event?

☐1.Yes ☐ 2. No

3. Have you ever taken alcohol during a new year celebration?

□1.Yes □ 2. No

4. Have you ever taken alcohol at a wedding event?

□1.Yes □ 2. No

5. Have you ever taken alcohol during annual harvest festival?

□1.Yes □ 2. No

6. Have you ever taken alcohol at a religious event?

□1.Yes □ 2. No

b) Drinking and Smoking Status

1. Have you drunk alcohol in the last 30 days?

□ 1.Yes □2. No

2. Do you smoke?

□ 1.Yes □2.No

3. Where do you usually drink?

□1. At home □2. Friend house □ 3. Dormitory

□ 4. School □5. Other...............

4. Are there alcohol shops in the neighborhood around your house?

□1. Yes □ 2. No

5. Are there alcohol shops in your school’s neighborhood?

☐1.yes ☐ 2. no

6. 10. Have you ever been sent to buy alcohol?

☐1.Yes ☐ 2. No

Part 4 Alcohol Knowledge

Question	Agree	I don't know	Disagree
1. Drinking alcohol could lead to road traffic accidents			
2. Alcohol drinking could cause of liver problems			
3. Alcohol drinking could lead to many noncommunicable diseases			
4. Alcohol improves sleep quality			
5. Alcohol is one of the narcotic drugs			
6. Drinking alcohol might lead to a mental problem			
7. Drinking alcohol might lead to criminal activities			

8. Drinking alcohol in pregnancy can affect the featus			
9. Thai law forbids children under 18 to buy or drink alcohol			
10. Thai law prohibits driving after drinking alcohol			

Part 5 Attitude towards alcohol consumption

Attitude towards alcohol	Strongly agree	agree	Not sure	Disagree	Strongly Disagree
1. Drinking alcohol symbolizes being a real man					
2. It is easy to make friends if one drinks alcohol					
3. Drinking alcohol makes health better					
4. Drinking alcohol is a symbol of maturity					
5. Drinking alcohol can reduce stress					
6. Drinking alcohol can reduce anxiety					

7. Drinking alcohol can reduce pain					
8. Drinking alcohol makes one look smart and attractive					
9. Drinking alcohol is completely normal for teenagers					
10. Everyone has the right to drink alcohol					

Part 6 Stress assessment

Questions	Never	Almost never	Sometimes	Fairly often	Often
1.In the last month, how often have you been upset because of					

something that happened unexpectedly?					
2. In the last month, how often have you felt that you were unable to control the important things in your life?					
3. In the last month, how often have you felt nervous and stressed?					
4. In the last month, how often have you felt confident about your ability to handle your personal problems?					
5. In the last month, how often have you felt that things were going your way?					
6. In the last month, how often have you found that you could not cope with all the things that you had to do?					

7. In the last month, how often have you been able to control irritations in your life?					
8. In the last month, how often have you felt that you were on top of things?					
9. In the last month, how often have you been angered because of things that happened that were outside of your control?					
10. In the last month, how often have you felt difficulties were piling up so high that you could not overcome them?					
